# Interpretable prediction of brain activity during conversations from multimodal behavioral signals

**DOI:** 10.1371/journal.pone.0284342

**Published:** 2024-03-21

**Authors:** Youssef Hmamouche, Magalie Ochs, Laurent Prévot, Thierry Chaminade

**Affiliations:** 1 International Artificial Intelligence Center of Morocco, University Mohammed VI Polytechnique, Rabat, Morocco; 2 LIS UMR 7020, CNRS, Aix Marseille Université, Université de Toulon, Marseille, France; 3 LPL UMR 7309, CNRS, Aix Marseille Université, Marseille, France; 4 INT UMR 7289, CNRS, Aix Marseille Université, Marseille, France; Kochi University of Technology, JAPAN

## Abstract

We present an analytical framework aimed at predicting the local brain activity in uncontrolled experimental conditions based on multimodal recordings of participants’ behavior, and its application to a corpus of participants having conversations with another human or a conversational humanoid robot. The framework consists in extracting high-level features from the raw behavioral recordings and applying a dynamic prediction of binarized fMRI-recorded local brain activity using these behavioral features. The objective is to identify behavioral features required for this prediction, and their relative weights, depending on the brain area under investigation and the experimental condition. In order to validate our framework, we use a corpus of uncontrolled conversations of participants with a human or a robotic agent, focusing on brain regions involved in speech processing, and more generally in social interactions. The framework not only predicts local brain activity significantly better than random, it also quantifies the weights of behavioral features required for this prediction, depending on the brain area under investigation and on the nature of the conversational partner. In the left Superior Temporal Sulcus, perceived speech is the most important behavioral feature for predicting brain activity, regardless of the agent, while several features, which differ between the human and robot interlocutors, contribute to the prediction in regions involved in social cognition, such as the TemporoParietal Junction. This framework therefore allows us to study how multiple behavioral signals from different modalities are integrated in individual brain regions during complex social interactions.

## Introduction

Investigating the causes of brain activity during natural social interactions is a difficult problem given that multiple cognitive processes are at play in such complex behaviors. Moreover, brain activity follows non-linear dynamics potentially influenced by factors that are difficult to measure, such as internal thoughts and other psychological factors. However, in order to better understand brain-behavior relationships in natural interactions, it is necessary to evaluate which behavioral event, and more importantly, which combination of such events, significantly influences local brain activity.

In this article, we tackle the problem of finding dependencies between neurophysiological time series in Regions Of Interest (or ROIs, which are intermediate functional units between neurons and the whole brain, corresponding to patches of cortical surface measuring several *mm*^3^). The analysis framework can be described with three main steps. We (*i*) use a non-invasive neuroimaging technique (here, fMRI) to record human brain activity during behaviors that are complex and uncontrolled behaviors (except for the specificities of fMRI recording such as laying supine in a scanner and avoiding head movements), as well as the behavior of the participants across multiple modalities, such as audio or video, and other changes in the environment the participants are interacting with, all of which are synchronized with the fMRI acquisition; then (*ii*) time-series describing high-level features are created from these raw recordings using knowledge from cognitive neuroscience that informs us about which behaviors can be extracted; finally (*iii*) machine learning methods are used to find relationships between the recorded fMRI signal and the behavioral features or multimodal combinations of such features. To validate this analysis framework, we then focus on uncontrolled conversations as an example of of complex social interaction, and on brain areas involved in speech perception on the one hand, and areas known to respond to behaviors related to social cognition in controlled experimental focusing on different sensory modalities on the other hand.

Testing relationships between time series can be performed using causality tests. Those tests are generally used to test relationships between one or multiple variables and a target variable. Many of those tests are based on prediction models, such as the Granger causality test and its alternatives [1]. The problem here is that we have a large set of multimodal predictive variables and we do not know which subset of variables we have to include in our models given the large number of possible combinations. The challenge is then, first, to find the subset of behavioral features that has an impact on a given neurophysiological time-series, and then to test their prediction. Therefore, we propose to first build a dynamic model in which the prediction of brain activity is coupled with the selection of features required for the prediction, and then predict brain activity on the basis of these features identified in terms of their ability to predict brain activity. An advantage of this approach is that the model can be used not only to detect relationships between variables, but also to predict local brain activity on the basis of behavioral features.

The majority of existing approaches test *a priori* hypotheses between brain activity and behavior [2–5], and rely on multivariate regressions to handle the problem of brain activity prediction based on behavior. Here, we propose to use advanced machine learning algorithms to predict the neurophysiological response based on recorded behaviors without relying on strong *a priori* hypotheses, allowing us to identify new relationships between specific aspects of complex behaviors on the one hand, and neurophysiology on the other hand. In practice, we distinguish two independent processes. First, several multimodal features are first extracted from raw behavioral signals. Second, the smallest set of features that have significant impact on the prediction of localized brain activity recorded with fMRI are identified in a second step.

Regarding the implementation, for (*i*), we use an existing corpus of uncontrolled conversations recorded with 24 participants, providing synchronized neurophysiological (fMRI) and behavioral signals [6]. This corpus is unique in the sense that participants’ behavior is unconstrained and therefore different from classical fMRI datasets generally acquired in highly controlled conditions. It includes human-human interactions (HHI) and human-robot interactions (HRI), which allows the analysis of the variability of relations between local activity and behavior not only in terms of the brain area under investigation, but also of the social context of the interaction operationalized by the nature on the conversational agent, human or artificial [7]. With regards to (*ii*), a number of recent publications investigating this corpus explain how high-level behavioral features have been extracted from raw recordings, either conversational features extracted from raw audio recordings after transcription and annotation [8], or visual features extracted from the video recordings of the interlocutor [9]. Other features used here rely on the recording of the eye movements of the participant and an exhaustive list of all raw recordings can be found in Rauchbauer *et al.*, 2020 [10].

In the current paper we mainly focus on the machine learning aspect of the framework (*iii*) that has been implemented for predicting fMRI brain activity based on multimodal raw signals of human-human or human-robot conversation. It brings two major contributions:

1. A *methodology for predicting discretized fMRI responses from behavioral signals of bidirectional conversation* is presented in the section Analysis Framework.
2. The *identification of dependencies between behavior and brain activity in specific areas during uncontrolled conversations* is presented in section Implementation.

In summary, we propose a novel analysis framework based on prediction of brain activity to elucidate relationships between brain activity, recorded with fMRI, and behavior, when the latter is not controlled experimentally. In addition to predicting local brain activity, the framework provides weight for the behavioral variables used for the prediction allowing interpretation of the findings in terms of brain-behavior relationships. The framework is applied to a unique corpus of human-human and human-robot conversations, shedding light on multimodal integration in key brain regions involved in social cognition such as the TemporoParietal Junction. As the next section will show, we have not found related work using a similar prediction approach to untangle brain-behavior relationships.

## Related work

Prediction using multiple modalities is a challenging task due to the diversity of the available signals to process. Classical prediction models are simpler in the sense that the input features belong to the same modality, and thus have similar structures. Concerning multimodal prediction, many problems arise, such as how to synchronize signals of different types and frequencies, how to represent data from each modality, and the fusion methodology to use for including all signals into one prediction model.

### Multimodal approaches

There are several real applications that involve multimodal signals to predict a given feature. Multimodal data are very useful in emotion recognition. For instance, a system can be trained to predict depression using audio, visual, and linguistic features [11]. Similarly, emotional states can be classified based on multimodal signals including audio, video, and physiological sensor signals [12]. In both papers, the approaches used are based on extracting multiple features from each modality, then fusing them in one model that performs a classification task based on the extracted features. This approach seems very logical since it enables explaining the results from the variables extracted so as to make interpretation possible. On the other hand, the approach that consists in building a one-step prediction model using the raw data as input may be efficient in terms of prediction accuracy, but lacks interpretability [13]. Multimodal data are also very common in the field of human social interaction. A multimodal approach has been proposed to predict back-channel feedback related to bidirectional human-human interaction [14]. These back-channels represent signs indicating the continuity of the interaction. The model used is probabilistic and it is based on Hidden Markov Models or Conditional Random Fields. The model takes as input three types of features, the prosody, the spoken words, and the eye gaze coordinates.

For more details about multimodal approaches, an important survey about multimodal approaches has been compiled [15]. It contains a general discussion of multimodal strategies for learning prediction models from information with different sources. The authors discuss the main and general steps for multimodal machine learning, and present several real applications involving multimodal data. They describe a set of fundamental steps for building a machine learning multimodal system, where the most important step is data representation, which is related to extracting and summarizing useful features from the different modalities. Other steps are also discussed regarding coordinating between the modalities, aligning and fusing them in order to make predictions. Those steps are very important, but each application requires a specific processing depending on the underlying modalities and the predictive variables.

### Brain activity prediction

Regarding the issue of predicting brain activity based on behavior, several approaches have been proposed in the literature. Some authors investigate the effect of adding visual information to auditory speech signals on the activity of auditory cortex areas [16]. The results show a significant increase in the activation of the studied regions of interests (ROIs) based on ANOVA. In another work [2], the fMRI neural activation associated to semantic processing is predicted based on a large text data-set. The brain regions studied are in the sensorimotor cortex. The model used consists in transforming the text into semantic features, then building a regression model that expresses the fMRI brain activity as a linear combination of semantic features. The authors show a prediction accuracy of 0.62 or higher, but on each participant independently. This issue has also been addressed with a multi-subject approach, by concatenating data of multiple participants. For example, it has been attempted to predict voxel activity from cortical areas, measured via the BOLD (Blood Oxygen Level Dependent) signal based on the speech signal [5]. The data used has been collected from an fMRI experiment on 7 subjects. The methodology adopted is based, first, on constructing semantic features from natural language analysis, then, dimensionality reduction using PCA (Principal Component Analysis) is applied to reduce the number of predictive variables, and a model is learned based on multiple linear regression with regularization in order to predict the BOLD signal. Finally, the obtained prediction results and the principal components of the predictive variables are both combined to classify brain areas according to the semantic features categories. Other types of behavioral signals have been investigated by evaluating the effect of a single feature on brain activity. For example, speech reaction time has been used to predict activity in specific brain regions [3]. The acoustically-derived vocal arousal score [17] is used to predict the BOLD signal using the Gaussian mixture regression model [4]. The authors can predict the BOLD signal in the posterior parietal cortex based on eye movement data using a multivariate regression model [18]. More general approaches have been tried to predict the brain activity of various areas using different types of signals at the same time. For example, correlations can be analyzed using linear regression between the BOLD signal and behavioral features computed from observed facial expressions, speech reaction time, and eye-tracking data [19].

### Multimodal approaches for brain activity prediction

In the related work presented above, dependencies between behavior and specific functional brain areas have been investigated. However, only one or few modalities have been taken into account. In addition, the methods used are generally based on correlation analysis or multiple regression. However, finding relevant predictive features using feature selection techniques with machine learning methods, such as prediction models based on artificial neural networks, can be particularly relevant for this research question. In this article, we propose a framework that consists in extracting high level features from raw multimodal behavioral data consisting of audio, video and eye-tracking recordings, then applying feature selection and prediction with different classifiers to predict discretized neurophysiological signals in circumscribed brain regions from multimodal behavioral signals.

## Analysis framework

### Overview

**Fig 1:**
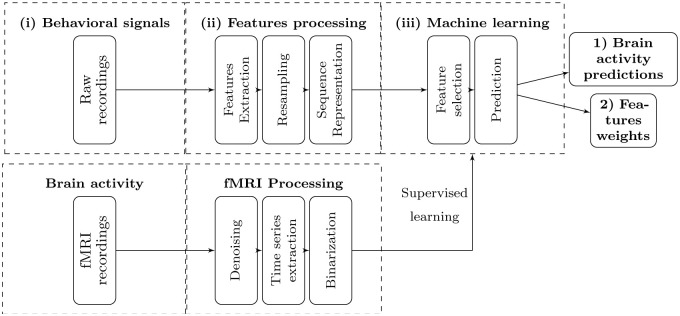
An illustration of the analysis framework. It is composed of three main steps. The first one (*i*) corresponds to the recording of raw input signals. The second step (*ii*) is the features preparation. In this step, high-level and interpretable features are extracted from input signals, then resampled and sequenced. The final step (*iii*) consists in training/testing machine learning models to predict brain activity time series and allow us to identify the relative weights of the the extracted features used in predicting the target variable.

The analysis framework presented is rooted in a meta-model using machine learning tools which allows, first, to predict brain activity from multimodal behavioral signals recorded in complex and uncontrolled behaviors, and second to quantify the relative weights assigned to the behavioral features extracted from raw recordings for this prediction. The output of the framework consists of the predictions of regional brain activity and the features selected for the prediction, and in particular the importance score of these selected features. The importance of the features is generally missing in most of the existing work, which merely identify features that trigger the activation of a brain area [3, 4, 17, 18]. Another, oft neglected, output is when the activity cannot be predicted, given the brain region of interest, the experimental condition(s) under scrutiny, and the features used for the prediction. The proposed framework is illustrated in Fig 1. In this section, we present its main steps.

The first step is to extract features from raw input signals for each modality separately in order to construct time series that will be used as predictive variables. These time-series necessitate resampling in order to have the same number of observations than the input signals recorded with different frequencies. Finally, the predictive variables also need to be restructured as sequences, since we are trying to predict the next pattern of brain activity based on the past values of the behavioral features. Then, feature selection methods and prediction models are applied to predict the discretized BOLD signals, as well as to find the smallest subset of features leading to the best possible predictions for each brain area and experimental context. Thus, the system does not require *a priori* hypotheses about the relationship between brain activity and behavior. Instead, it allows to find new relationships between complex uncontrolled behaviors on the one hand, and brain activity on the other hand, which can be interpreted from a neuroscience and social cognition point of view in order to identify complex multimodal cognitive mechanisms. The system uses a new prediction model based on temporal dynamics to predict the discretized brain activity time series based on the behavioral features.

### (*i*) Behavioral signals recordings

The analysis framework we propose has been developed for a specific corpus, namely fMRI recordings of unconstrained conversation between the participant and a fellow human or a conversational robotic head [6]. It may be applicable to other neurophysiological modalities, like EEG or MEG, but such generalization of the approach would require additional assumptions and processes that are beyond the focus of the present report. In the corpus analyzed later here, fMRI was recorded while participants were behaving as naturally as possible, *i.e.*, within the limits of fMRI recording (lying supine in an MRI scanner and required to keep the head as still as possible) but not constrained by rigorously controlled experimental conditions, in contrast to classical experimental approaches developed for the study of the neural bases of human cognition. Such corpora, including fMRI recordings during unconstrained behaviors, are likely to become increasingly available as it becomes clear that the neural bases of complex behaviors cannot be simply conceived as the addition or concatenation of simple behaviors but entail complex interplay between cognitive processes and their neural correlates. In social cognition, this point has been at the core of “second-person neuroscience” as proposed by Leonhard Schilbach and colleagues that is “*based on the premise that social cognition is fundamentally different when we are in interaction with others rather than merely observing them*” [20]. In other words, natural social interactions should not be simplified as series of largely independent sequences of action observation and execution, but should take into account behavior more thoroughly, with all its complexity and dynamics. A case has clearly been made for the study of the neural bases of social interactions in the most natural setting possible, given the already numerous constraints of fMRI. Given the focus on social interactions through conversation in the current case (see section Implementation), audio recordings of the speech produced by the two interlocutors best describe their behavior during the interaction. But it should be emphasized that the methodology presented can be used with other input signals. For example, one can imagine that emphasis be put on other sensory modalities if natural multisensory integration is the focus of interest, or on visual landmarks if natural spatial navigation is under scrutiny. In other words, the behavioral signals recorded synchronously with the fMRI data depends largely on the type of natural human behavior under investigation.

### (*ii*) Feature extraction

Integrating multimodal signals within the same deep learning architecture is a delicate task compared to classical unimodal architectures. With multimodal signals, different networks must coordinate to make efficient and robust predictions. Our idea is to transform raw input modalities into interpretable sequences, *i.e.*, features that describe behaviors that can be described verbally, such as a binary time-series describing whether one person is speaking or not. Such a strategy allows us to have a unified classification network that not only works for all modalities together, but can also work with any single one.

The aim of the (ii) feature extraction step is to compute high-level features from the raw recordings. There are three main reasons for using higher-order behavioral features. First, raw recordings are in a format that is difficult to introduce directly into machine learning models. This can be illustrated by comparing the content of raw audio files recorded during, *e.g.*, a conversation, to the linguistic transcriptions of these recordings. The former are saved as pulse-code modulations at 44kHz, that do not match the fMRI acquisition of 1.2 sec repetition time, while transcriptions encode the semantic content of the social interaction and are closer to the ∼1Hz frequency resampling of the fMRI sequences. Second, raw sound signals are likely to be correlated with the response of the primary auditory brain areas while transcriptions that encode the semantic content of the social interaction are more likely to be associated with higher-order linguistic areas (see for example [8]). The same is true of the correlation between the raw video recordings, that correspond to what participants are watching, and response in early visual areas, or with raw gaze recordings and activity in brain regions associated with motor control of eye movements. A similar approach was used to distinguish the brain correlates associated with the processing of low- *vs.* high-level, semantic representations of the emotions extracted from voice signals, showing a shift from early feed-forward processing of stimulus categories to a later processing of the salience of the stimuli [21].

A final and major reason for choosing extracted features over raw signal is their explainability [22], defined as the ability of “a system [to] deliver or contain accompanying evidence or reason(s) for outputs and/or processes”. It is indeed straightforward to make sense of a variable such as “Speech activity” (that defines whether the participant or the interlocutor is speaking) than the raw pulse-code modulation of the audio signal. Furthermore, this step is based on domain knowledge, that is, it makes use of knowledge accumulated in specific domains of cognitive neuroscience, such as linguistics or visual processing.

Altogether, our approach is to use time-series describing behavioral features that are known to affect brain activity as input to the machine learning models. Importantly, our approach is easily amenable to improvements as domain knowledge itself progresses and provides new high-level features that can be extracted from raw signals and used for the prediction step, therefore allowing to evaluate their changes in predictive power. Iteratively, such an approach could help refine the types of representations used in the brain during natural behaviors. As a result, an obvious limitation, but also a strength, of the approach, is that the current results only provide a snapshot constrained by the raw signals used and the features extracted in the current implementation, but that can later be compared with new features when they become available and are added to this extraction step.

### (*iii*) Machine learning

#### Feature selection

Feature selection is performed on the variables representing the temporal sequences of the extracted recorded features. The goal here is to identify the most relevant set of variables in terms of the prediction accuracy for each variable that will be predicted. In the following, we detail three different methods that we have used for feature selection.

*Wrapper feature selection method*. This method uses the prediction model itself to perform the selection of the appropriate variables. First, the prediction model is executed with all features. Then, the top *k* features are selected based on the weights provided by the model. This method is simple in terms of implementation, but its main drawback is that the selected features are specific to the prediction model used, which means that if we change the model, the selected features can also change.

*Ranking based on mutual information*. The filter method selects variables without the need of a prediction model. It ranks variables based on their Shannon mutual information (MI) with the target variable. The Shannon MI works only for discrete variables. For example, the bivariate MI between two variables *X*, *Y* can be expressed as follows:

$$\begin{array}{c} I(X;Y)=\sum_{x,y}P(x,y)log(\frac{P(x,y)}{P(x)P(y)}) \end{array}$$ (1)

In our case, we can not use formulation (1) directly because some features are continuous. Two solutions are possible to overcome this problem, the first and most common one consists in discretizing the continuous features, and then to use the Shannon MI. The second one is to use a continuous estimation of the Shannon MI, as proposed in [23]. The authors present an estimation of MI based on *k*-nearest neighbors approach, which relies an idea similar to the one for estimating the continuous entropy [24]. This MI estimator can be expressed as follows:

$$\begin{array}{c} \hat{I}\left(X;Y\right)=\Gamma \left(k\right)+\Gamma \left(n\right)-\frac{1}{n}\sum_{i=1}^{n}\left(\Gamma \left(n_{x}\left(i\right)+1\right)+\Gamma \left(n_{y}\left(i\right)+1\right)\right), \end{array}$$ (2)

where Γ is the Gamma function, *n* is the number of observations of *X* and *Y*, *k* is the number of neighbors to consider, *n*_*x*_(*i*) is the number of points where the distance from *x*_*i*_ is strictly less than *d*_*i*_/2, where *d*_*i*_ is the distance from *X*_*i*_ to its *k*^*th*^ neighbor.

*Clustering-based method*. This method is another filter-based feature selection method. It corresponds to an extension of the previous method in the sense that it considers the mutual information between predictive variables in addition to their mutual information with the target variable. It first groups close predictive variables into clusters using the *k* *-medoids* algorithm based on the principle of maximizing mutual information within clusters and minimizing mutual information between clusters. Then, it selects one variable from each cluster based on the mutual information with the target variable. This method is designed to work with multimodal data, since the fact of grouping variables before ranking them could be a relative solution to the problem of dependencies between variables that belong to the same group.

#### Predictions

**Fig 2:**
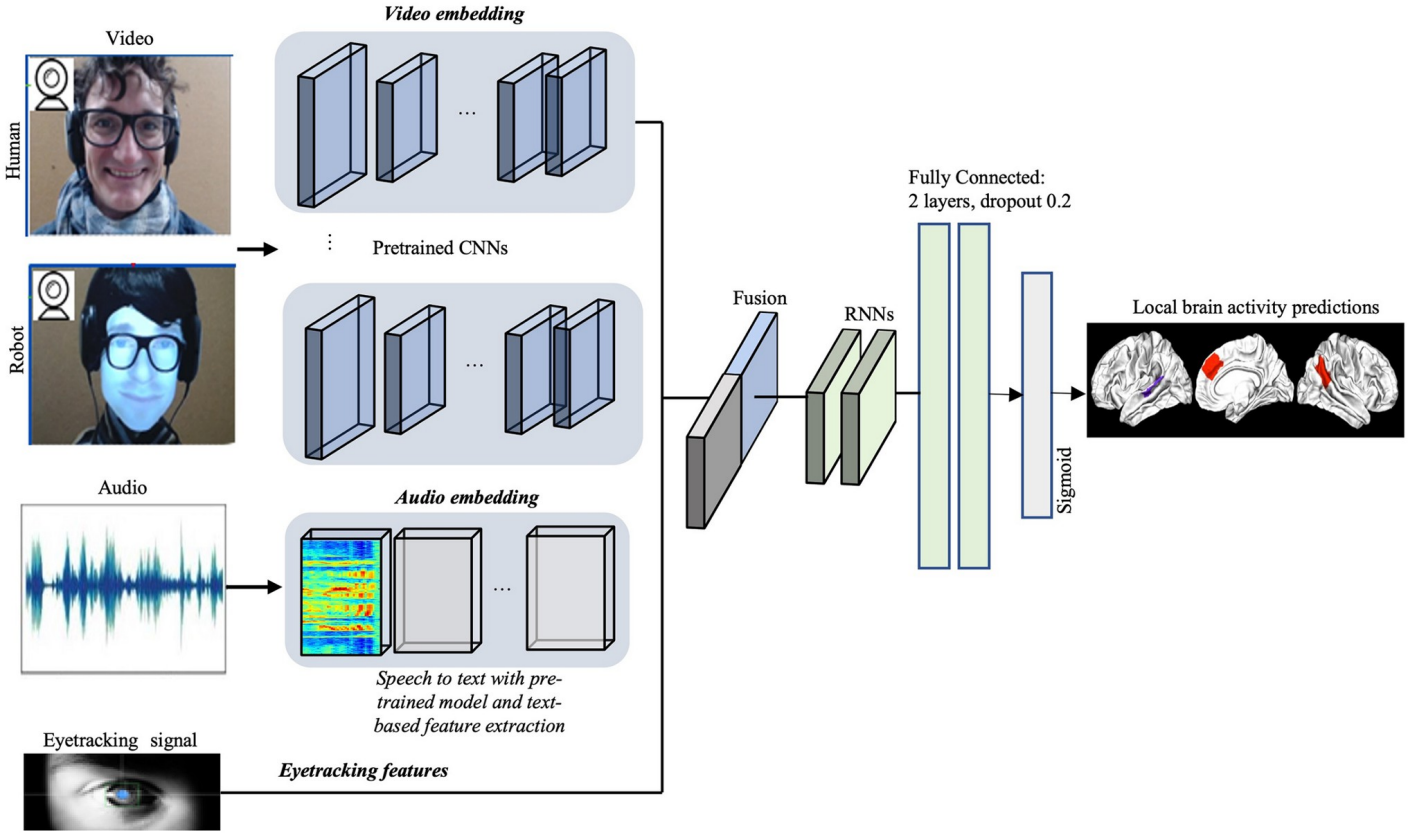
Illustration of our multimodal prediction approach. Feature extractions are performed based on specific methods and pre-trained models. Then the obtained time series are re-sampled and concatenated, and then they are fed to a network composed of LSTM and fully connected layers.

Our approach is based on a multimodal fusion. This step creates a shared representation of the features irrespective of their individual modality. The sequenced behavioral features obtained previously are fed into deep network classifiers and then a fully connected layers. The classifiers used belong to two types. The first type includes the Random Forest (RF), Support Vector Machine (SVM), and Logistic Regression (LReg). The second type includes models based on neural networks including a fully connected network and an LSTM (Long Short Term Memory) network. For both networks, the backpropagation (through time for LSTM) is applied to train the network over 50 iterations using the stochastic gradient descent (SGD) algorithm. The multimodal architecture with the LSTM network is illustrated in Fig 2.

To measure the robustness of our predictions, we added a baseline classifier that generates random predictions (named Rand in the rest of this article). We trained this classifier after tuning a parameter representing the predictions generation strategy. Three strategies are considered: a stratified strategy generates predictions regarding the distribution of the training data, a uniform strategy generates predictions uniformly, and a third strategy is based on the most frequent class. The strategy that provides the best prediction results in the training stage is the one that is kept for testing the baseline classifier. The classifications metrics used are the F-score and the Recall. The recall is the percentage of examples classified as positive, among the total number of positive examples, while the F-score is more balanced since it considers both false positives and false negatives. Note that Precision can also be inferred from the results since the F-score is the weighted average of Precision and Recall.

Finally, Student’s *t*-tests are performed to test the equality (null hypothesis) of the average F-scores between the best and the baseline classifiers obtained in the training step. Student’s *t*-tests are the most recommended and used statistical tests to compare machine learning models [25].

## Implementation

Here, we describe how the general framework presented in the previous section was used to analyze a specific data-set. More precisely, the proposed analysis framework is used to (i) predict brain activity from multimodal signals of bidirectional human-human and human-robot conversational interactions (respectively HHI and HRI), and (ii) to identify dependencies between brain activity in Regions Of Interest (ROIs) known to be involved in the phenomenon under investigation (conversational social interaction) and high-level features extracted from raw behavioral signals. The data-set is fully relevant with the issues the analysis framework is supposed to address, namely physiological and multi-modal behavioral recordings performed during complex behaviors, between which we want to identify dependencies. The complex behaviors under scrutiny are unconstrained conversations between participants and a fellow human and a robot. Inputs of the framework are, on the one hand, brain activity measured in fMRI via the BOLD signal, and on the other hand, behavioral signals of bidirectional conversations between a participant whose brain activity is scanned inside the fMRI machine and an interlocutor, either a human or a robot, located outside the fMRI machine. Three types of conversational signals are taken into account: speech, facial and head movements, and eye movements.

### (*i*) Corpus

The data used in this work were collected with fMRI recordings described in previous work [6], and are publicly available [26]. Each participant’s recordings (n = 24 in the sample used here) consists of four sessions, each containing six conversations of 60 seconds, three with a human and three with a conversational robot in alternating order. An *advertising campaign* provides a cover story to make sure that the participants are unaware that the actual focus of the experiment is to record a corpus of uncontrolled social interactions: participants are told that they should guess what is the message carried by images in which fruits appear either as *superheroes* or *rotten*. Each conversation between the participant and either a confederate of the experimenter or a Furhat conversational robot [27] (controlled by the confederate in a *Wizard-of-Oz* mode, unbeknownst to the participant), is about one single image of the purported *advertising campaign*. The project received ethical approval from the Comité de Protection des Personnes (CPP) Sud-Marseille 1 (approval number 2016-A01008–43). Written consent was obtained from all participants. The input raw data consist of 3 types of signals: video of the interlocutor (human or robot), speech (raw audio recordings and manual transcriptions) of both the participant and the interlocutor, and eye-tracking recordings of the participant. Note that videos of the participants could not be recorded as they were inside the scanner during the fMRI experiment.

### fMRI data preparation

Data from fMRI requires processing. First, whole brain recording is processed to remove as much of the noise it contains as possible. It is then parcellated in regions of interest to summarize activity of functionally homogeneous areas. The mean data is extracted in each region of interest, single trials extracted from the continuous recordings of the session, and finally binarized. In addition, the BOLD response lags from the behavioral sources with a delay modelled by the hemodynamic response function that needs to be taken into account.

#### Processing fMRI signals

Standard fMRI acquisition procedures were used, described in detail in Rauchbauer et al., 2019 [6]. BOLD signal 3-dimensional images are recorded in the whole brain every 1.205 seconds (repetition time). Standard SPM12 preprocessing procedures are used [28], including correction for time delays in slice acquisition (“slice timing”), image realignment, magnetic field inhomogeneities correction, normalization to the standard MNI space using the DARTEL [29] procedure for coregistration of individual participants’ anatomy, and finally spatial smoothing with a 5-mm full-width half-maximum 3-dimensional Gaussian kernel. Extraction of the BOLD signal in regions of interest is performed using the conn toolbox [30], and includes several denoising procedures. Firstly a linear detrending using a high-pass filter with a threshold of 128 seconds, then using realignment parameters to calculate nuisance regressors related to participants’ movement during scanning, thirdly, taking heartbeat and breathing recordings to remove physiological artifacts with the PhysIO toolbox [31], and finally extracting BOLD signal in the white matter and cerebrospinal fluid and using the 5 first eigenvariates of the time-series as nuisance representing signal fluctuations in non-cortical brain tissues.

#### Brain regions of interest (ROIs)

**Fig 3:**
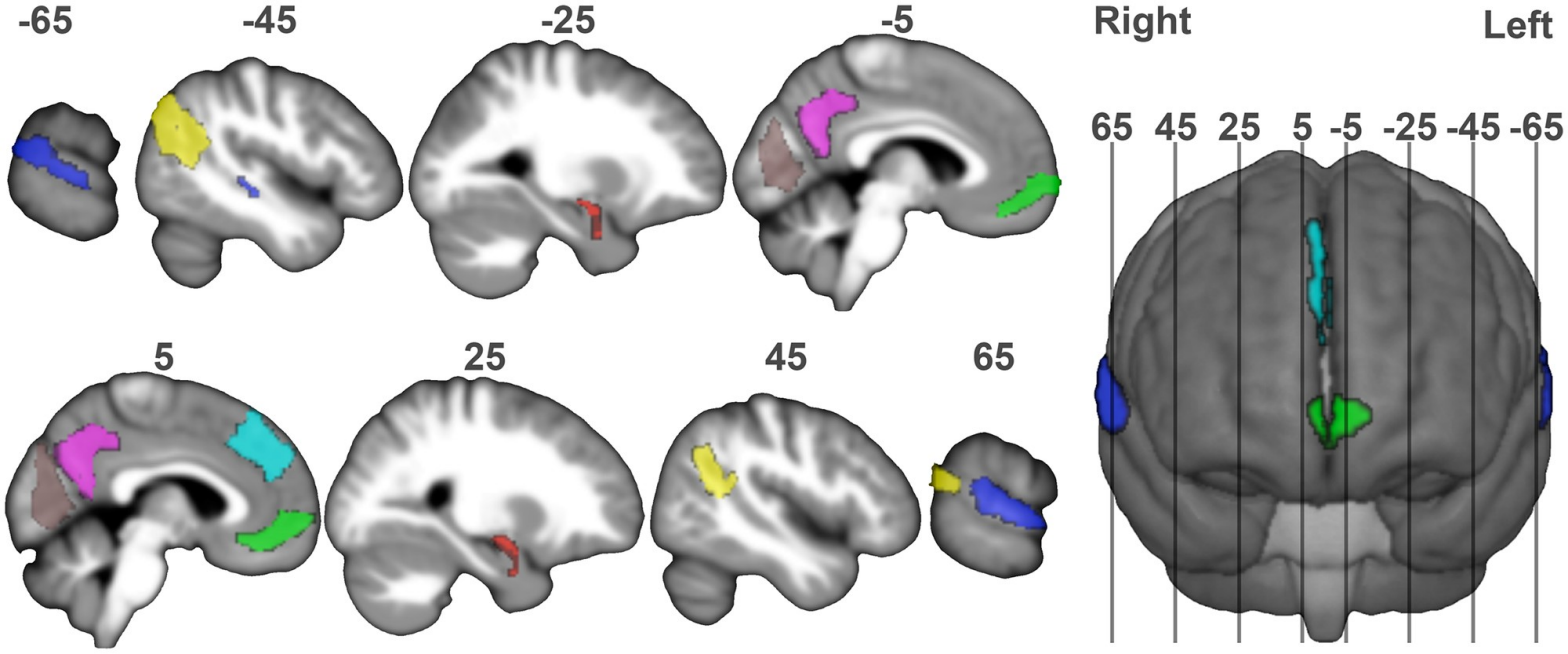
Regions of interest (ROIs) under investigation superimposed to sagittal sections of the average of the participants’ brains normalized to the Montreal Neurological Institute (MNI) template space. Section locations (in *mm* from midbrain section, negative for the left hemisphere) are indicated by numbers above the sections and on the reference three-dimensional render seen from the front on the right panel. ROI order as in Table 2 for clarity: Primary Visual Cortex (V1): brown; Superior Temporal Sulcus (STS): Blue; TemporoParietal Junction (TPJ): Yellow; Precuneus (Pre): Pink; Amygdala (Amy): Red; VentroMedial PreFrontal Cortex (VMPFC): Green; DorsoMedial PreFrontal Cortex (DMPFC): cyan.

A 275-area parcellation based on functional and anatomical connectivity patterns (https://atlas.brainnetome.org/bnatlas.html, [32]) defines ROIs for the whole brain. Continuous time-series (385 time points) are extracted for each ROI and each session and participant representing the mean activity within the ROI after denoising. In this paper, we focus on specific regions (Fig 3 and Table 1) involved in social cognition (TemporoParietal Junction TPJ, Precuneus Pre) including speech perception (in the caudal superior temporal sulcus region STS) and emotional processing (Amygdala Amy and ventral Medial PreFrontal Cortex vMPFC). Two different types of control ROIs were also included, one corresponding to the white matter, where signal fluctuations are not supposed to reflect neuronal information processing and shouldn’t be predictable [3], and another in the Primary Visual Cortex where predictions should rely on visual information instead of auditory or social information. Finally, the case of the dorsal Medial PreFrontal Cortex (dMPFC) is of particular interest, as it has been associated both with low-level motor control [33] and high-level social cognition [34], and it is expected that the features best able to predict activity in this region will allow us to disentangle these two interpretations, visuomotor control of eye movements *vs.* social cognitive processes.

**Table 1:** The regions of interest (ROIs).

Abbreviations	Brain areas	Brainnetome atlas
l,r V1	left and right Primary Visual Cortex	191 192
l,r STS	left and right Superior Temporal Sulcus	75 76
l,r TPJ	left and right TemporoParietal Junction	143 144
l,r Pre	left and right Precuneus	153 154
l,r Amy	left and right Amygdalae	213 214
l,r VMPFC	left and right VentroMedial PreFrontal Cortex	41 42
r DMPFC	right DorsoMedial PreFrontal Cortex	12
WM	White Matter	

#### Binarization

Importantly, the raw BOLD is a continuous measure. A binarization of the signal into 0 (inactive) and 1 (active) is required as our approach consists in predicting whether a brain region is active or not. Such binarization of the BOLD signal has been used repeatedly when machine learning approaches are applied to fMRI signals (*e.g.* [35–39]). Here, we use a binarization method proposed by Ostu et al. [40] reproducing previous approaches based on filtering the BOLD signal of each brain area into two states (active *vs.* non active) using its average over the time points of a trial for thresholding.

#### Compensating for the hemodynamic response delay

**Fig 4:**
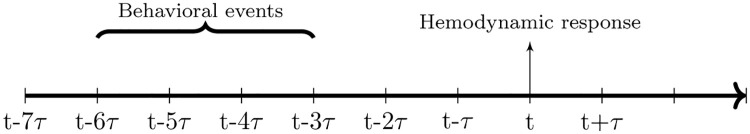
The time delay corresponding to the hemodynamic response to behavioral events is taken into account in the model by considering four consecutive behavioral time points happening between 7.2 and 3.6 seconds before the time *t*, where the hemodynamic response is evaluated.

The BOLD signal follows the Hemodynamic Response Function (HRF), which characterizes the signal to a single behavioral event [41]. This function peaks with a delay around five seconds that can vary depending on the brain area, the participant, as well as other factors that are not well known. To handle this variability, we express the discretized bold signal at time *t* based on a sequence of 4 previous consecutive observations of behavioral features which span the duration between *t* − *τ*_1_ and *t* − *τ*_2_, where *τ*_1_ = 7.2*s* and *τ*_2_ = 3.6*s* (Fig 4).

The number of observations in every sequence depends on the re-sampling rate of behavioral features with respect to the target variable. In the current study, we re-sample them with the same frequency of the BOLD signal, that is, one observation every *τ* = 1.2*s*. We also tried to re-sample the BOLD signal based on interpolation to have an observation each 0.6*s* so as to double the number of temporal features. The performance results show that this re-sampling does not improve the predictions while requiring more computational time. Therefore, we focus in this work on the same re-sampling rate for both BOLD and behavioral features by resampling the latter.

Let *y*_*t*_ be the discretized time series associated to the BOLD signal, and *x*_*t*_ = {*x*_1,*t*_, *x*_2,*t*_, …, *x*_*k*,*t*_} are *k* behavioral time series, representing the predictive features, where *x*_*i*,*t*_ is the *i*^*th*^ variable at time *t*. We use the following notation to represent the sequences of behavioral variables in a concise way:

$$\begin{array}{c} x_{i}^{t-\tau_{1}:t-\tau_{2}}=(x_{i,t-6\tau },x_{i,t-5\tau },x_{i,t-4\tau },x_{i,t-3\tau }). \end{array}$$ (3)

As mentioned before, we aim at predicting *y* at time *t* based on a sequence of *x* between *t* − 8*s* and *t* − 4*s*, with a time-step *τ* = 1.2*s*. This is a temporal classification problem, and with the previous notation, our dynamic model can be expressed as follows:

$$\begin{array}{c} y_{t}=f(x_{1}^{t-\tau_{1}:t-\tau_{2}},\cdots ,x_{k}^{t-\tau_{1}:t-\tau_{2}})+e_{t}, \end{array}$$ (4)

where *f* is function of the model, and *e*_*t*_ represents its error vector.

### (*ii*) Behavioral features processing

The aim of feature extraction is to compute high-level features from multimodal raw behavioral data, that may describe specific social and conversational factors involved in a conversation. Our feature extraction approach is based on interpretable features that are computed automatically from raw multimodal signals. From the raw recordings, we extract high-level features described in Table 2. They are also described with additional details in Rauchbauer *et al.*, 2020 [10]. The extraction process is in itself performed using deep learning models that rely on multiple types of networks:

- Computer vision-based networks for video processing: emotion recognition, face and eye movements, and most of the facial features in Table 2.
- Classical and recurrent neural networks for the audio and text data: sentiment analysis, semantic and structural features from text, spectral features.
- Classical Time series analysis for eyetracking signals.

**Table 2:** Description of the extracted multimodal features.

	Feature	Description	Details
**Linguistic features**	SpeechActivity	The interlocutor speaking?	Based on time-aligned IPU transcript.
	Overlap	Both interlocutors speaking?	*idem*.
	TurnLatency	Time to take the turn	*idem*.
	SpeechRate	Speaking speed	*idem*.
	Laughter	Laughter occurrences	*idem*.
	FilledPauses	Filled-Pauses occurrences	Based on word-level time-aligned transcripts: *‘euh’, ‘heu’, ‘hum’, ‘mh’*.
	Feedback	Conversational Feedback occurrences	*idem*: *‘oui’ (yes), ‘ouais’ (yeah), ‘non’ (no), ‘ah’, ‘d’accord’ (right), ‘ok’* + Laughters.
	DiscourseMarkers	Occurrence of words used to keep speech organized	*idem*: *‘alors’ (so), ‘mais’ (but), ‘donc’ (therefore), ‘et’ (and), ‘puis’ (then), ‘enfin’ (finally), ‘parce que’ (because), ‘ensuite’ (after)*.
	SpokenParticles	Occurrence of (final) spoken particle items	*idem*: *‘quoi’, ‘hein’, ‘ben’, ‘bon’ (well), ‘mais’ (but), ‘bref’ (in short)*.
	Interpersonal	Merge of inter-personal linguistic features	Merge of (Filled-pauses, Feedback, Discourse Markers, Spoken Particles and Laughter).
	TypeTokenRatio	Lexical richness measure	Based on time-aligned transcript: (number of different tokens) / (total number of tokens).
	LexicalRichness	Lexical richness measure [42]	Based on time-aligned transcript: (number of adjectives + number of adverbs) / (total number of tokens).
	Polarity & Subjectivity	Sentiment analysis metrics [43]	Based on time-aligned transcript, and a pre-trained KNN classifier.
**Facial features**	Head-Tx, Head-Ty, Head-Tz	Head translation	Based on head pose estimated using Openface [44].
	Head-Rx,Head-Ry, Head-Rz	Head rotation	*idem*.
	Head-T-energy	Kinetic energy of head translation	*idem*.
	Head-R-energy	Kinetic energy of head rotation	*idem*.
	AU-mouth	Sum of facial movements related to mouth	Based on Facial Action Units (AUs) existence detected by Openface library [44].
	AU-eyes	Sum facial movements related to eyes	*idem*.
	AU-all	Sum of all action units	*idem*.
	Direct-gaze	Percentage of direct gaze direction of the conversant	Based in gaze coordinates provided by Openface library [44].
	‘Neutral’, ‘Happiness’, ‘Sadness’, ‘Surprise’, ‘Fear’, ‘Anger’, ‘Disgust’	Emotions displayed by the interlocutor	Based on a pre-trained CNN classifier.
	Smiles	Smile’s detection	Based on a CNN classifier from Opencv library.
**Eyetracking**	Saccades	Occurrence of Saccades	Based on gaze coordinates of the participant, recorded using the Eyelink1000 system.
	Gaze-speed	Gaze Speed	*idem*.
	Gaze-movement-energy	Gaze movements energy	*idem*.
	Face-looks,Eyes-looks,Mouth-looks	Number of looks in face, eyes and mouths *respectively*	Based on participant’s gaze coordinates and interlocutor’s detected landmarks.

Facial features are directly extracted from the video of the interlocutor, speech features are extracted from manual transcriptions of the participant and interlocutor’s recorded conversations, and eye-tracking features are extracted from participant’s gaze recorded inside the fMRI scanner. The features are constructed as time series for each conversation. For example, in the case of speech, we analyzed Inter-Pausal Units (IPUs are speech blocks of one speaker bounded by pauses [45]) by IPU (for example to compute the lexical richness) or word by word (*e.g.*, to compute Feedback and Discourse Markers). For videos, Openface 2.0 toolkit [44] is used to detect facial action units, landmarks, head pose estimation, and gaze coordinates. Eyetracking coordinates of the participant are recorded using the Eyelink1000 system. We added other features characterizing where the participant is looking (Face, Eyes, Mouth), by combining the detected landmarks points of the conversant and the gaze coordinates.

In total, more than 40 features were extracted. Table 2 contains the names of the extracted features per modality with a description of how each feature is extracted. For more details, all features are available online https://github.com/Hmamouche/NeuroTSConvers, and are also described in Rauchbauer *et al.* 2019 [6]. The extracted features require further processing in order to homogenize their structure. This processing includes a resampling the obtained features with respect to the frequency of the fMRI signal and a concatenation of the time series of all trials and participants.

To make the results more interpretable, we created higher-order features by grouping individual features (see Table 2) into meta-features that each represents one aspect of behavior that is relevant for social cognition (see Table 3). The underlying assumption is that it is not the importance score of each individual feature that is relevant, but the importance scores of all features pertaining to similar aspects of social cognition. For example, all individual features measuring head movements of the interlocutor (translations, rotations, energy) are pooled together as “dynamics of the interlocutor head movements” or HeadDynamics-I. Importantly, meta-features were defined after results from original features were calculated, and correspond to the sum of importance weights of these original features. They were not used as such during the (*iii*) Predictions calculation.

**Table 3:** Regrouping the extracted features into social meta-features (suffix “-P” for the participant, and “-I” for the interlocutor).

Meta-features	Original features
SpeechDynamics-I	SpeechActivity-I, Overlap-I, SpeechRate-I
SpeechDynamics-P	SpeechActivity-P, Overlap-P, SpeechRate-P
SpeechComplexity-I	LexicalRichness-I, TypeToken-Ratio-I
SpeechComplexity-P	LexicalRichness-P, TypeToken-Ratio-P
SpeechSocial-I	FilledBreaks-I, Feedbacks-I, Discourses-I, Particles-I, Laughters-I, Interpersonal-I, Polarity-I, Subjectivity-I, Turn-Latency-I
SpeechSocial-P	FilledBreaks-P, Feedbacks-P, Discourses-P, Particles-P, Laughters-P, Interpersonal-P, Polarity-P, Subjectivity-P, Turn-Latency-P
HeadDynamics-I	Head-Rx-I, Head-Ry-I, Head-Rz-I, Head-Tx-I, Head-Ty-I, Head-Tz-I, Head-T-energy-I, Head-R-energy-I
FaceDynamics-I	AU-all-I, AUs-mouth-I, AUs-eyes-I, Neutral-I
FaceEmotions-I	Happiness-I, Sadness-I, Surprise-I, Fear-I, Anger-I, Disgust-I, Smiles-I
GazeDynamics-P	Gaze-speed-P, Gaze-movement-energy-P, Saccades-P
GazeSocial	Face-looks-P, Mouth-looks-P, Eyes-looks-P, Direct-gaze-I

### (*iii*) Predictions

The framework proposed in Analysis Framework is a general meta-model that can be used in a variety of ways depending on the data-set under investigation. Now, we describe how it was applied to the data-set described above.

Human-human and human-robot data are evaluated separately in order to compare results from the two conditions. For each condition, the obtained data consist of 13248 observations. We fix the training set to 18 participants from 24, and we apply the ADASYN algorithm [46] on this set to address the problem of imbalanced data. This algorithm generates new observations by considering the distribution of the data.

Then, we performed a 9-fold-cross-validation on training data in order to find the appropriate parameters of the classifier based on the F-score measure. We choose 9 folds instead of the classical 10 folds because we use data from 18 participants out of 24 in the training set and we want to evaluate the models on data of participants unseen by the models in order to avoid over-fitting. The models are then tested on data of 6 participants, that is, 25% of all data. We apply one training-test pass directly. Student’s *t*-tests are performed to test the equality (null hypothesis) of the average F-scores between the different models and the baseline classifier obtained in the training step via the 9-fold cross-validation, with a significance threshold of *p*≤0.05.

## Results

In this section, we present prediction results. They include performance scores of the used classifiers, as well as interpretable results about the most relevant features for each ROI.

### Statistically significant predictions

F-score and recall measures calculated for each condition (human-human and human-robot interaction), each region of interest as well as the different models used are given in the supplementary material (Tables 7 and 8 in S1 File for the F-score and the Recall, respectively). Here, we focus on the models that yielded the best F-scores obtained for both conditions in all ROIs, reported in Table 4 the recall score. Prediction of ROI activity for HHI are significant for all ROIS except the lV1, lAmy and lVMPFC, while for HRI there are only 5 areas with significant prediction F-scores (lSTS, rSTS, rPre, lTPJ, and rTPJ).

**Table 4:** F-scores obtained by the best model in the different ROIs for the HHI (Human-Human Interaction) and HRI (Human-Robot Interaction) conditions. The ***p***-values are provided for the best model. Bold indicates significant ***p***-values at the threshold (0.05).

ROIs	F-scores HHI			F-scores HRI		
	Best	Rand	*p*-value	Best	Rand	*p*-value
lV1	0.58	0.50	*0.143*	0.56	0.50	*0.999*
rV1	**0.59**	0.53	***0.005***	0.58	0.51	*0.221*
**lSTS**	**0.71**	0.50	***≤0.001***	**0.69**	0.51	***≤0.001***
**rSTS**	**0.71**	0.49	***≤0.001***	0.71	**0.52**	***≤0.001***
**lTPJ**	**0.61**	0.50	***≤0.001***	**0.59**	0.51	***0.036***
**rTPJ**	**0.64**	0.50	***≤0.001***	**0.63**	0.54	***≤0.001***
lPre	**0.60**	0.51	***≤0.001***	**0.57**	0.50	*0.503*
**rPre**	**0.62**	0.50	***≤0.001***	**0.57**	0.50	***0.036***
lAmy	0.59	0.49	*0.081*	0.61	0.51	*0.114*
rAmy	**0.58**	0.48	***0.004***	0.64	0.51	*0.560*
lVMPFC	0.55	0.49	*0.190*	0.54	0.50	*0.216*
rVMPFC	**0.58**	0.50	***0.015***	0.54	0.51	*0.858*
rDMPFC	**0.66**	0.54	***≤0.001***	0.66	0.54	*0.157*
WM	0.54	0.51	*0.436*	0.51	0.51	*0.796*

### Interpretable prediction results

In this part, we present detailed results concerning the selected features that lead to the best prediction for each brain area. We also show their importance scores in order to discuss the impact of each meta-feature on the predictability of the brain regions of interest under investigation.

The results include recall score and the set of the best predictive meta-features and their respective scores to reach the prediction. Note that a threshold of 10% is used for reporting meta-features to focus on the most important ones. Tables 5 and 6 contain these results for HHI and HRI respectively.

**Table 5:** Meta-features (with importance score ≥ 0.10) used to significantly predict ROIs activity in HHI. Recall score is obtained from the best model classifier (see Table. 8 in S1 File).

ROI	Recall score	Meta-features	Importance scores
rV1	0.63	FaceDynamics-I	0.45
		GazeDynamics-P	0.38
		HeadDynamics-I	0.16
lSTS	0.71	SpeechDynamics-I	0.85
		SpeechSocial-I	0.12
rSTS	0.70	SpeechDynamics-I	0.52
		SpeechDynamics-P	0.21
		GazeSocial	0.15
lTPJ	0.62	SpeechDynamics-P	0.33
		SpeechDynamics-I	0.25
		HeadDynamics-I	0.21
		GazeSocial	0.11
rTPJ	0.70	SpeechDynamics-P	0.33
		SpeechSocial-P	0.67
lPre	0.63	GazeDynamics-P	0.58
		HeadDynamics-I	0.16
		FaceDynamics-I	0.15
		SpeechDynamics-P	0.11
rPre	0.63	HeadDynamics-I	0.38
		SpeechDynamics-P	0.17
		FaceDynamics-I	0.15
		GazeSocial	0.11
rAmy	0.63	SpeechDynamics-P	0.26
		SpeechDynamics-I	0.23
		SpeechSocial-I	0.15
		SpeechComplexity-I	0.15
rVMPFC	0.61	GazeDynamics-P	0.45
		HeadDynamics-I	0.34
rDMPFC	0.72	SpeechDynamics-P	1.00

**Table 6:** Meta-features (with importance score ≥ 0.10) used to significantly predict ROIs activity in HRI. Other details as in Table 5.

ROI	Recall score	Meta-features	Importance scores
lSTS	0.69	SpeechDynamics-I	1.00
rSTS	0.70	SpeechDynamics-I	0.75
		SpeechDynamics-P	0.25
lTPJ	0.62	GazeDynamics-P	0.19
		SpeechDynamics-I	0.16
		FaceDynamics-I	0.15
		SpeechDynamics-P	0.14
		SpeechSocial-I	0.13
rTPJ	0.67	Speech Dynamics-P	1.00
rPre	0.62	SpeechDynamics-P	0.88
		GazeDynamics-P	0.11

## Discussion

### Prediction in cortical regions of interest *vs.* white matter

Results indicate that the proposed analysis framework is able to predict brain activity higher than chance in a number of cortical regions of interest (ROIs) but not in white matter. This results served as a control comparison given that the white matter is not known to display large BOLD responses in response to external events [3]. Even if such response can be found with very specific settings, it is unlikely to be associated with the subtle and complex behavioral features we are investigating here. In addition, we used a whole-brain white matter mask to extract the white matter signal, so that any localized response is lost by the averaging of signal coming from different fibers. Signal from the white matter was also regressed out during the denoising of the fMRI data. This absence of prediction in the brain white matter confirms that the significant results we found in a number of ROIs are due to correct prediction and not spurious explanations.

### Effect of experimental conditions on prediction

Importantly, predictions are significantly greater than chance for both HHI and HRI in the Superior Temporal Sulcus (STS) and TemporoParietal Junction (TPJ) bilaterally. The former was selected as being an area strongly devoted to language perception, as this region of the cortex contains “voice patches”, regions responding specifically to the perception of voices [47]. Therefore, perceiving speech from others is likely to activate these patches in a simple on/off fashion. Indeed, the highest F-scores are obtained in these regions in both hemispheres and both interaction conditions. Furthermore, on the left hemisphere, dominant for language, the interlocutor’s speech (SpeechDynamics-I) is the only predictor (with an importance score of 1) used to reach a recall score of 0.69 in the HRI condition (see Table 6). There is ongoing speculation about a more general involvement of this part of the cortex in social cognition, for example in visual perception [48]. The second meta-feature used for prediction on the right STS region in HRI is the speech produced by the participant (SpeechDynamics-P, with an importance score of 0.25), indicating a complex interaction between producing and perceiving speech in the non-dominant hemisphere. SpeechDynamics-P is also an important meta-feature for predicting rSTS activity in HHI, but other meta-features associated with social aspects of the interaction are also predictive of STS activity for HHI: SocialSpeech in the left hemisphere and SocialGaze in the right hemisphere, indicating the importance of social processes when interacting with a human, but not a robot. In other words, while in both HRI and HHI, the second predictor for the right STS is the speech of the participant, these two speech-related meta-features are completed with social ones bilaterally for the human interlocutor, but not for the robot. Altogether, this results derived from a uncontrolled discussion with a natural and artificial agent fit largely with expectations, with specialized left STS for speech perception, right STS being also involved in speech production, and non-verbal social behavioral meta-features when the interlocutor is a human.

#### Response in regions of interest (ROIs) associated with unimodal processes

The right Primary Visual Cortex (rV1) receives the input of retinal information in the cortex. Indeed, its response is explained by the head and facial movements of the interlocutor as well as the participant’s eye movements, all related to the visual input. It was not predicted in HRI, possibly because the robot’s movements were very limited compared to the human’s. The DorsoMedial PreFrontal Cortex region is only associated with speech production in HHI, which is more consistent with its role in the motor control of language production [33] than in mentalizing [34]. Its absence in HRI could also be explained by the fact that speech was also lessened when participants interacted with the robot compared to the human [10].

#### Response in regions of interest (ROIs) associated with social cognition

A second series of observations can be made for areas associated with social cognition in which we obtained significant F-scores. These areas include rTPJ, lTPJ, and rPre for both HHI and HRI, and VMPFC, Amy and rDMPFC for HHI only. First, their prediction scores are smaller than those of the left and right STS. Then, in a number these areas, notably the left TemporoParietal Junction (TPJ) for both HHI and HRI and the left and right Precuneus (lPre & rPre), right Amygdala (rAmy) for HHI only, several meta-features, including different types of signals, including social ones, are combined to provide significant predictions. These two remarks support the hypothesis that these areas are associating more complex multimodal signals than the STS. Given that we acknowledge that defining features relevant for social cognition is exploratory, we do expect activity in these areas to be less predictable. Interestingly in HRI, and despite the closeness of these two areas on the right hemisphere’s cortical surface (see Fig 3), speech activity of the participant is the only predictor for the TPJ, while the speech activity of the interlocutor is the only meta-feature for STS. This result argues against the possibility that non-specific relations could be identified due to the proximity of our regions of interest on the cortex. It should also be noted that a number of additional meta-features unrelated to speech are recruited to significantly predict activity in the left TPJ for both human and robot interlocutors. In particular, the importance of visual signals (head movements in HHI, facial movements in HRI) highlights a complex combination of different sensory modalities while experimental paradigms that focus on one modality only, for example reading that requires visual processing [49], are usually preferentially associated with right hemisphere responses. It is possible that complex associations of multiple modalities are not correctly captured by classical experimental paradigms focusing on more controlled, and unimodal, aspects of social cognition.

Predictions in areas involved in emotional aspects of social cognition, VentroMedial PreFrontal Cortex and Amygdala, yield very similar results: activity can only be predicted for human-human interaction and in the right hemisphere. Several meta-features are used for predictions, including SpeechSocial-I for the amygdala and eye and head movements from the participant and interlocutor respectively for the VentroMedial PreFrontal Cortex.

#### Validation of the analysis framework

Altogether, results from higher-order associative areas are double-sided. On the one hand, they confirm to a large extent the ability to predict activity from complex associations of meta-features. This is for example the case of the activity in the Precuneus, known a more complex response in HHI than HRI in this experiment [50], that can only be predicted in HHI and the right hemisphere. But on the other hand, it is not obvious new relationships could be discovered when relations between multimodal behavioral features and brain activity are limited—either by the limited association between the behavior and cognition in the case of emotional processing for example in the left hemisphere, or by the poverty of the interaction in the case of robot.

This final remark is confirmed by the *p*-values of the statistical test performed of the mean of F-scores of the best classifier and the baseline (random predictions generator) using the Students *t*-test. Results show that the *p*-values obtained for HRI are greater than those of HHI for all brain areas investigated. Interacting with humans, that is, having a social interaction, is more natural than interacting with an artificial agent, and in particular the robot used in this experiment. This is associated with differences in brain activity associated with the two conditions available in the corpus, as was found not only with a simpler analysis of the same corpus [6] but also with other comparisons of HHI and HRI [51]. There is, in particular, a difference in the way mental states of the interlocutor are represented by the participant depending on whether the former is a natural or an artificial agent [52]. It was therefore expected that the higher-order mental representations, that correspond to meta-features associated with social processing, would be more strongly involved when the interlocutor is a human than when it is a robot, as the present results show, in particular for the TemporoParietal Junction bilaterally in condition HHI.

## Limitations

Several limitations of the analytical framework proposed in this paper must be highlighted. First, it is intriguing that activity in all areas could not be predicted with the features used. Several explanations can be proposed, and three are particularly relevant. First, it could be that the non-linear associations between the neural signals used and behavior preclude finding any relation between the two despite the use of an approach supposedly able to reveal such non-linear relations. Second, it is possible that some of the areas are associated with processes that are not directly related to behavior, such as internal thoughts, that cannot be used to form behavioral features used to predict brain activity. Finally, and more interestingly, we acknowledge that additional behavioral features inspired by knowledge coming, for example, from domains that were not at all considered in this analysis, could be the most relevant features to predict brain activity in some brain regions. We expect that adding additional features should improve not only prediction scores in areas significantly predicted, but also allow prediction in additional areas. An example of such features could be emotional variables extracted from physiological recordings, such as heart-rate variability or pupil size, that were not considered in the current analysis.

Another important limitation is the finding that different machine learning approaches yielded the best predictions depending on the regions of interest and/or experimental conditions. While we chose to select the best models for later analysis of the behavioral features weights used for the prediction, such inconsistency is puzzling, as one would have expected that similar mathematical approaches to brain-behavior relations would have worked across regions. It should still be noticed, as visible in Tables 9 and 10 in S1 File, that in many cases the scores obtained by the different models were rather similar, though quantifying this similarity is beyond the objectives of the current paper.

At present, it is difficult to address the issue of false positives. Though the results reported here make sense given the literature, as argued in the discussion, our approach does not provide us with a test to assess the statistical significance of the weights of individual behavioral features, and we cannot exclude some of them as being false positives. Furthermore, some behavioral features could be related not to the behavior after which they were designed but to another behavior (such as hidden mental states like internal thoughts) that is temporally correlated to the observable behavior. This limitation can be overcome by adding more features and precising their relation to mental processes in other corpora, thus using reproducibility as a hallmark of real relations.

Finally, some of the parameters were chosen arbitrarily, and though we attempted to ground these choices in the literature, it may be that in the current framework these were not optimal. An example of such choice is the threshold used for binarization of the BOLD signal, based on its average for each region and trial, that could be performed at the whole brain level before parcellation, or the choice of the ADASYN algorithm to handle unbalanced data, that may not match the peculiarities of the neuroimaging signal. Further investigations are needed to fine-tune these parameters not only with the current corpus, but also as other corpora are investigated with the same analytical framework.

## Conclusion

In this paper, we present a new analysis framework grounded in the prediction of fMRI brain activity from behavioral signals and identifying dependencies between them in uncontrolled experimental settings. Evaluation of this framework is made on a corpus comprising human-human and human-robot conversations of 24 participants. We focused on brain areas involved in speech perception and in social interaction. We obtained predictions significantly better than those obtained with a random classifier in many of the brain regions investigated. The obtained dependencies confirm existing hypotheses about the relationship between circumscribed aspects of behaviors and functional brain areas, but also allows to identify new relations pertaining to multimodal integration of behaviors in brain areas involved in higher-order aspects of cognition. The framework’s output provides new results, by finding the best possible model, the associated subset of relevant features and a quantification of their impact for each brain area. In addition, they allow to compare the difference between brain activation in the cases of human-human and human-robot interaction in terms of the predictive behavioral features for each brain area. The next step of this work is to test the framework on all brain areas. Understanding why certain regions cannot be predicted is a puzzling finding requiring further investigation, and in particular the addition of new behavioral features in the future.

## Supporting information

S1 File (PDF)
