# Supplementary material for: Interpretable prediction of brain activity during conversations from multimodal behavioral signals
Source: PLoS One. 2024 Mar 21;19(3):e0284342. doi: 10.1371/journal.pone.0284342 (PMC10956754; doi:10.1371/journal.pone.0284342)
Supplement: S1 File — (PDF) [file pone.0284342.s001.pdf]

Supplementary information

Detailed prediction results

The detailed prediction F-scores and recalls with all the models tested in this analysis tested on human-human and human-robot data-sets are provided in Tables 7 and 8.

Table 7. The F-scores obtained by each model.

ROIs	F-scores HHI						F-scores HRI					
	LReg	LSTM	FNN	RF	SVM	Rand	LReg	LSTM	FNN	RF	SVM	Rand
lV1	**0.58**	0.57	0.56	0.57	0.57	0.50	0.52	0.53	0.53	**0.56**	0.55	0.50
rV1	0.59	0.59	0.55	**0.60**	**0.60**	0.53	0.56	0.57	0.56	**0.58**	**0.58**	0.51
lSTS	**0.71**	0.70	0.70	**0.71**	0.68	0.50	0.68	0.68	0.66	**0.69**	0.68	0.51
rSTS	0.70	0.69	0.69	**0.71**	0.68	0.49	0.70	**0.71**	0.70	0.67	0.63	0.52
lTPJ	0.58	0.58	0.59	**0.61**	0.60	0.50	**0.59**	0.58	0.55	0.58	0.56	0.51
rTPJ	0.63	0.63	0.61	**0.64**	**0.64**	0.50	0.62	0.62	0.59	**0.63**	**0.63**	0.54
lPre	0.55	0.58	0.55	0.60	0.59	0.51	0.52	0.54	0.53	**0.57**	0.56	0.50
rPre	0.57	0.58	0.58	**0.62**	0.60	0.50	0.49	0.49	0.50	0.56	**0.57**	0.50
lVMPFC	0.47	0.50	0.55	0.54	0.55	0.49	0.47	0.52	**0.54**	**0.54**	**0.54**	0.50
rVMPFC	0.54	0.55	0.57	0.57	**0.58**	0.50	0.47	0.45	0.49	**0.54**	**0.54**	0.51
rDMPFC	**0.66**	0.60	0.57	0.65	0.65	0.54	**0.66**	0.64	0.58	0.62	0.64	0.54
lAmy	0.58	0.55	0.54	**0.59**	0.53	0.49	0.55	0.58	0.55	0.58	**0.61**	0.51
rAmy	0.53	0.57	**0.58**	0.57	0.54	0.48	**0.64**	0.61	0.58	0.61	0.61	0.51
WM	0.47	0.46	0.49	**0.54**	0.53	0.51	0.46	0.46	0.46	**0.51**	0.38	**0.51**

Table 8. The Recall scores obtained by each model.

ROIs	Recalls HHI						Recalls HRI					
	LReg	LSTM	FNN	RF	SVM	Rand	LReg	LSTM	FNN	RF	SVM	Rand
lV1	**0.62**	**0.62**	0.57	0.58	0.61	0.49	**0.59**	**0.59**	**0.59**	0.56	0.55	0.49
rV1	**0.63**	0.59	0.55	0.60	0.62	0.52	**0.63**	0.61	0.60	0.60	0.58	0.50
lSTS	**0.71**	**0.71**	**0.71**	0.70	0.68	0.49	**0.69**	0.68	0.67	**0.69**	0.68	0.50
rSTS	**0.70**	0.70	0.69	**0.71**	0.67	0.48	0.70	**0.71**	0.69	0.66	0.63	0.51
lTPJ	**0.62**	**0.62**	0.60	0.61	0.60	0.49	**0.62**	0.59	0.56	0.58	0.55	0.50
rTPJ	**0.66**	0.64	0.63	0.64	0.65	0.49	**0.67**	**0.67**	0.63	0.63	0.65	0.52
lPre	**0.63**	0.62	0.60	0.62	0.59	0.50	**0.62**	0.55	0.54	0.57	0.59	0.49
rPre	**0.63**	0.61	0.59	**0.63**	0.62	0.49	0.61	**0.62**	**0.62**	0.58	0.58	0.49
lVMPFC	0.57	0.56	**0.58**	0.54	0.54	0.49	0.59	0.52	0.56	0.54	**0.57**	0.50
rVMPFC	**0.61**	0.58	0.59	0.58	0.58	0.49	0.57	0.57	**0.61**	0.54	0.57	0.50
rDMPFC	**0.72**	0.68	0.57	0.65	0.65	0.51	0.69	**0.71**	0.61	0.61	0.66	0.51
lAmy	**0.65**	0.54	0.55	0.59	0.52	0.48	**0.66**	0.63	0.59	0.63	0.62	0.50
rAmy	**0.61**	0.59	0.59	0.57	0.59	0.48	**0.64**	0.60	0.58	0.61	0.61	0.50
WM	**0.61**	**0.61**	0.60	0.54	0.54	0.51	**0.61**	**0.61**	**0.61**	0.51	0.43	0.50

Table 9. Model and feature selection yielding the best results on ROIs with significant predictions for HHI conditions.

ROI	Model	Feature selection	F-score	Recall score
lV1	SVM	Model-rank	0.60	0.62
lSTS	RF	Model-rank	0.61	0.71
rSTS	RF	MI-rank	0.71	0.70
lTPJ	RF	Model-rank	0.61	0.61
rTPJ	SVM	k-medoids	0.64	0.65
lPre	RF	Model-rank	0.60	0.62
rPre	RF	MI-rank	0.62	0.63
rVMPFC	SVM	Model-rank	0.58	0.58
rDMPFC	LSTM	MI-rank	0.66	0.72
rAmy	FNN	k-medoids	0.58	0.59

Table 10. Model and feature selection yielding the best results on only ROIs with significant predictions for HRI conditions.

ROI	Model	Feature selection	F-score	Recall score
lSTS	RF	Model-rank	0.69	0.69
rSTS	LSTM	MI-rank	0.71	0.71
lTPJ	LReg	Model-rank	0.59	0.62
rTPJ	SVM	k-medoids	0.63	0.65
rPre	SVM	MI-rank	0.57	0.58
